# Stochastic Entropy Solutions for Stochastic Nonlinear Transport Equations

**DOI:** 10.3390/e20060395

**Published:** 2018-05-23

**Authors:** Rongrong Tian, Yanbin Tang

**Affiliations:** School of Mathematics and Statistics, Hubei Key Laboratory of Engineering Modeling and Scientific Computing, Huazhong University of Science and Technology, Wuhan 430074, China

**Keywords:** nonlinear transport equation, stochastic (strong) entropy solution, uniqueness, existence, 60H15, 35R60

## Abstract

This paper considers the existence and uniqueness of stochastic entropy solution for a nonlinear transport equation with a stochastic perturbation. The uniqueness is based on the doubling variable method. For the existence, we develop a new scheme of parabolic approximation motivated by the method of vanishing viscosity given by Feng and Nualart (J. Funct. Anal. 2008, 255, 313–373). Furthermore, we prove the continuous dependence of stochastic strong entropy solutions on the coefficient *b* and the nonlinear function *f*.

## 1. Introduction

In this paper, we consider the existence and uniqueness of the solutions to the nonlinear transport equation with a stochastic forcing:

(1) $$\begin{array}{c} \left\{\begin{array}{c} d\rho \left(t,x\right)+b\left(x\right)\cdot \nabla_{x}f\left(\rho \left(t,x\right)\right)dt=A\left(\rho \left(t,x\right)\right)dW_{t},\;\;t>0,\;x\in R^{d},\\ {\rho \left(t,x\right)|}_{t=0}=\rho_{0}\left(x\right),\;x\in R^{d}, \end{array}\right. \end{array}$$

where $W_{t}$ is a one-dimensional Wiener process on a stochastic basis ($\Omega ,F,P,{\left\{F_{t}\right\}}_{t⩾0})$ and A:R→R is a real valued function. f:R→R and $b:R^{d}\to R^{d}$ are Borel functions, and the initial data $\rho_{0}$ is non-random.

When ${\mathrm{div}}_{x}b=0$, then $b\left(x\right)\cdot \nabla_{x}f\left(\rho \left(t,x\right)\right)={\mathrm{div}}_{x}\left(b\left(x\right)f\left(\rho \left(t,x\right)\right)\right)$, the equation in (1) models the phenomenon of complex fluid mixing in porous media flows and other problems in mathematics and physics [1,2,3,4,5]. A particular application of this model involves two-phase fluid flow, which has been used to study the flow of water through oil in a porous medium [6,7]. For the porous media flows, the spatial variations of porous formations occur on all length scales, but only the variations at the largest length scales are reliably reconstructed from data available. The heterogeneities occurring on the smaller length scales have to be incorporated stochastically. Consequently, the flows through such formations are stochastic [8].

There has been an interest in studying the effect of stochastic force on the corresponding deterministic equations, especially on the existence and uniqueness. Most of papers focus on the following Cauchy problem:

(2) $$\begin{array}{c} \left\{\begin{array}{c} d\rho \left(t,x\right)+{\mathrm{div}}_{x}F\left(\rho \left(t,x\right)\right)dt=A\left(t,x,\rho \left(t,x\right)\right)dW_{t},\;\;t>0,\;x\in R^{d},\\ {\rho \left(t,x\right)|}_{t=0}=\rho_{0}\left(x\right),\;x\in R^{d}, \end{array}\right. \end{array}$$

where $W_{t}$ is a one-dimensional standard Brownian motion or a cylindrical Brownian motion, or a space-time Gaussian white noise.

The various well-posedness results have been established for the Cauchy problem (2). When d=1, the $L^{\infty }$ solution has been established in [9,10] for A=A(ρ) and A=A(t,x), respectively, under hypotheses that $\rho_{0}\in L^{\infty }$ and *A* has compact support. For general *A*, even for initial data $\rho_{0}\in L^{\infty }$, the solution is not in $L^{\infty }$ since the maximum principle is not available. Therefore, $L^{p}$ (1⩽p<∞) is a natural space on which the solutions are posed.

When A=A(x,ρ), the framework of $L^{p}$-solutions (2⩽p<∞) was first established by Feng and Nualart [11], but the existence was true only for d=1. These solutions were generalized to weak-in-time by Bauzet, Vallet and Wittbold [12], Biswas and Majee [13], and Karlsen and Storrøsten [14]. For any dimension d⩾1, the well-posedness of kinetic solutions was obtained by Debussche and Vovelle [15], and then the result was extended by Hofmanová [16]. Recently, due to the fact that uniform spatial BV-bound is preserved for problem (2) if *A* satisfies a Lipschitz condition, Chen, Ding and Karlsen [17] supplied a result on well-posedness of $\cap_{p⩾1}L^{p}$ solutions in $R^{d}$ for d⩾1. Furthermore, there are many papers devoted to the study of the Cauchy problem (2), such as the study on bounded domains [18,19,20], invariant measures [21,22], Lévy noises [23,24,25,26] and long time behaviors [27]. For more details in this direction for random fluxes, we refer the readers to [28,29,30,31].

When *F* depends explicitly on *x*, so far as we know, there are few research works on the Cauchy problem (2). Even though for the problem (1), there are still few works since that the presence of *b* will bring us some new difficulties on the proof of existence and uniqueness of solutions. Moreover, from the viewpoint of conservations laws and numerical simulations, $L^{\infty }$ is a natural space on which solutions are posed, how to get the boundedness of solutions is another difficulty. We would like to point out that there are two big difficulties arisen here. One is how to get the compactness of solutions for the viscosity equation, another is how to prove the boundedness of solutions. To overcome the first difficulty, we develop a new scheme of parabolic approximation, which sheds some new light on the method of vanishing viscosity. For the second difficulty, we use the Ito’s formula and the cut-off technique. We know that there are probably three classical methods to deal with the compactness of solutions for the viscosity equation so far when *F* is independent of *x*. The first is based upon Young’s relaxed measure [11,14], which is suitable to space-time Gaussian white noise. The second is to estimate the spatial BV-bound and temporal $L^{1}$-continuity [17], which is suitable to get the convergence of solutions for almost everywhere (t,x) and almost surely ω. The third is to use the kinetic formulation [15,16], which is suitable to cylindrical Brownian motion.

In this paper, we adapt the method given by [11,14], but there is a significant difference. The more important thing is that we obtain the continuity of solutions in the temporal variable. The arguments for problem (1) can be generalized to an equation in which the stochastic term is represented by

$$\begin{array}{c} \int_{z\in Z}A\left(x,\rho \left(t,x\right),z\right)W\left(t,dz\right), \end{array}$$

where *Z* is a metric space, and *W* is a space-time Gaussian white noise martingale random measure with respect to the filtration ${\left\{F_{t}\right\}}_{t⩾0}$, if one assumes in addition that *A* is Lipschitz continuous in *x*. Up to longer and more tedious calculations, the arguments for space-time Gaussian white noise is similar to problem (1). There is no new component except some minor changes, which is also similar to the proof given in [11]. To make the present proof more refined, we discuss the simple case and prove the existence and uniqueness of solutions to (1) in this paper. Encouraged and inspired by the definition given in [11], we first give a notion of stochastic entropy solution.

**Definition** **1.** *Let $|b|,\mathrm{div}b\in L_{loc}^{1}\left(R^{d}\right)$, $f\in C^{2}\left(R\right)$, A∈C(R), $\rho_{0}\in L^{1}\cap L^{\infty }\left(R^{d}\right)$. An ${\left\{F_{t}\right\}}_{t⩾0}$-adapted and $L^{2}\left(R^{d}\right)$-valued stochastic process ρ=ρ(t,x,ω) is said to be a stochastic entropy solution of (1), if*

*(i)*  *for every T>0 and every p∈[1,∞),*
  (3) $$\begin{array}{c} \rho \in C(\left[0,T\right];L^{p}\left(\Omega ;L_{loc}^{p}\left(R^{d}\right)\right)) \end{array}$$
  *and*
  (4) $$\begin{array}{c} \underset{0⩽t⩽T}{\sup}{∥\rho \left(t\right)∥}_{L^{1}\left(\Omega \times R^{d}\right)}+\underset{0⩽t⩽T}{\sup}{∥\rho \left(t\right)∥}_{L^{\infty }\left(\Omega \times R^{d}\right)}<\infty ; \end{array}$$
*(ii)*  *for every entropy pair (η,q), $(\eta \in C^{\infty }\left(R\right),\eta^{\prime \prime }⩾0,q\left(v\right)=\int^{v}\eta^{\prime }\left(s\right)f^{\prime }\left(s\right)ds$, $f^{\prime }\left(s\right)=df\left(s\right)/ds$), every nonnegative function $\varphi \in C_{0}^{2}\left(R^{d}\right)$ and every 0⩽s<t<∞,*
  (5) $$\begin{array}{cc} & \int_{R^{d}}\varphi \left(x\right)\eta \left(\rho \left(t,x\right)\right)dx-\int_{R^{d}}\varphi \left(x\right)\eta \left(\rho \left(s,x\right)\right)dx\\ ⩽ & \int_{s}^{t}\int_{R^{d}}{\mathrm{div}}_{x}\left(b\left(x\right)\varphi \left(x\right)\right)q\left(\rho \left(r,x\right)\right)dxdr+\frac{1}{2}\int_{s}^{t}\int_{R^{d}}\eta^{\prime \prime }\left(\rho \left(r,x\right)\right)A^{2}\left(\rho \left(r,x\right)\right)\varphi \left(x\right)dxdr\\ & +\int_{s}^{t}dW_{r}\int_{R^{d}}\eta^{\prime }\left(\rho \left(r,x\right)\right)A\left(\rho \left(r,x\right)\right)\varphi \left(x\right)dx,\;P-a.s., \end{array}$$
  *where the stochastic integral in the last term in (5) is interpreted in Itô’s sense.*

*Furthermore, stochastic entropy solution ρ is called a stochastic strong entropy solution if the below conditions hold:*

*(iii)*  *for each ${\left\{F_{t}\right\}}_{t⩾0}$-adapted $L^{2}\left(R^{d}\right)$-valued stochastic process $\tilde{\rho }\left(t,x,\omega \right)$, satisfying (3) and (4), we define $\tilde{\eta }$ through each entropy function η by*
  (6) $$\begin{array}{c} \tilde{\eta }\left(r,v,y\right):=\int_{R^{d}}\eta^{\prime }\left(\tilde{\rho }\left(r,x\right)-v\right)A\left(\tilde{\rho }\left(r,x\right)\right)\psi \left(x,y\right)dx, \end{array}$$
  *where r⩾0, v∈R, $y\in R^{d}$ and $\psi \in C_{0}^{2}\left(R^{2d}\right)$, there is a deterministic function D(s,t), such that*
  (7) $$\begin{array}{c} E\int_{R^{d}}[\int_{s}^{t}\tilde{\eta }\left(r,v,y\right)dW_{r}{]}_{v=\rho (t,y)}dy⩽E\int_{s}^{t}\int_{R^{d}}\partial_{v}\tilde{\eta }\left(r,v=\rho \left(r,y\right),y\right)A\left(\rho \left(r,y\right)\right)dydr+D\left(s,t\right); \end{array}$$
*(iv)*  *for each T>0, there exist partitions $0=t_{0}<t_{1}<\cdots <t_{n}=T$ such that*
  (8) $$\begin{array}{c} \lim_{\max(t_{i}-t_{i-1})\to 0}\sum_{i=1}^{n}D\left(t_{i-1},t_{i}\right)=0. \end{array}$$

We now state our main results. The first one is focused on the uniqueness.

**Theorem** **1.** *Let $f\in C^{2}\left(R\right)$, $\rho_{0}\in L^{1}\cap L^{\infty }\left(R^{d}\right)$ and*

(9) $$\begin{array}{c} b\in BV_{loc}\left(R^{d};R^{d}\right),\;\mathrm{div}b,\;\frac{\left|b(\cdot )\right|}{1+|\cdot |}\in L^{\infty }\left(R^{d}\right),\;A\in C^{\frac{1}{2}}\left(R\right). \end{array}$$

*Suppose that $\rho_{1}$ and $\rho_{2}$ are stochastic entropy solutions of (1), and one of them is a stochastic strong entropy solution. Then, for every t>0,*

(10) $$\begin{array}{c} E∥\rho_{1}\left(t\right)-\rho_{2}{\left(t\right)∥}_{L^{1}\left(R^{d}\right)}=0. \end{array}$$

**Remark** **1.** Compared with the uniqueness results given in [11,17], Theorem 1 is new since the 1/2-Hölder continuity of A is enough to ensure the uniqueness. Moreover, compared with the uniqueness result for stochastic differential equations in [32], the hypotheses of 1/2-Hölder continuity on A is optimal.

If *b*, *f* and *A* are more regular, we also have the following existence results.

**Theorem** **2.** *Let $f\in C^{2}\left(R\right)$ such that $f^{\prime }$ is bounded and f(0)=0. Assume that $b\in W^{1,\infty }\left(R^{d};R^{d}\right)$ and*

(11) $$\begin{array}{c} \rho_{0}\in L^{1}\cap L^{\infty }\left(R^{d}\right),\;A\in Lip\left(R\right),\;A\left(0\right)=0,\;and\;\exists \;N>0,\;A\left(u\right)=0,\;\forall \;\left|u\right|⩾N. \end{array}$$

*Then,*

*(i)*  (1) has a stochastic strong entropy solution.
*(ii)*  *Moreover, in addition $\rho_{0}\in BV\left(R^{d}\right)$, for every T>0, we have $\rho \in L^{\infty }\left(\left[0,T\right];L^{1}\left(\Omega ;BV\left(R^{d}\right)\right)\right)$ and there is a constant C depending only on ${∥b∥}_{W^{1,\infty }\left(R^{d}\right)}$ and $∥f^{\prime }{∥}_{L^{\infty }\left(R\right)}$ such that*
  (12) $$\begin{array}{c} \underset{0⩽t⩽T}{\sup}{E∥\rho \left(t\right)∥}_{BV(R^{d})}⩽{C(∥b∥}_{W^{1,\infty }\left(R^{d}\right)},∥f^{\prime }{∥}_{L^{\infty }\left(R\right)})∥\rho_{0}{∥}_{BV(R^{d})}. \end{array}$$

**Remark** **2.**

*(i)*  If divb=0, then f(0)=0 is not needed.
*(ii)*  For a general function A, even for initial data $\rho_{0}\in L^{\infty }$, the solution is not in $L^{\infty }$. To maintain the boundedness of solutions, additional assumptions on A should be added. Inspired by [9,10], we can suppose that A has compact support.

We now discuss the continuous dependence of the solutions on b,f and *A*. Some results for the continuity on *A* have established for the case of constant vector field *b* [17]. Here, we only give the continuous dependence of the solutions on *b* and *f*.

**Theorem** **3.** *Let ${\tilde{\rho }}_{0}\in L^{1}\cap L^{\infty }\left(R^{d}\right)$, $\rho_{0}\in L^{1}\cap L^{\infty }\cap BV\left(R^{d}\right)$, $b,\tilde{b}\in W^{1,\infty }\left(R^{d};R^{d}\right)$. $f,\tilde{f}\in C^{2}\left(R\right)$ such that $f^{\prime },{\tilde{f}}^{\prime }$ are bounded and $f\left(0\right)=\tilde{f}\left(0\right)=0$. A meets the assumption (11). Let ρ be the unique stochastic strong entropy solution of (1) and $\tilde{\rho }$ be the unique stochastic strong entropy solution of*

$$\begin{array}{c} \left\{\begin{array}{c} d\tilde{\rho }\left(t,x\right)+\tilde{b}\left(x\right)\cdot \nabla_{x}\tilde{f}\left(\tilde{\rho }\left(t,x\right)\right)dt=A\left(\tilde{\rho }\left(t,x\right)\right)dW_{t},\;t>0,\;x\in R^{d},\\ \tilde{\rho }{\left(t,x\right)|}_{t=0}={\tilde{\rho }}_{0}\left(x\right),\;x\in R^{d}. \end{array}\right. \end{array}$$

*For every T>0, there exists a constant C>0, which depends only on ${∥b∥}_{W^{1,\infty }\left(R^{d}\right)}$ , ${∥f^{\prime }∥}_{L^{\infty }\left(R\right)}$, ${∥{\tilde{f}}^{\prime }∥}_{L^{\infty }\left(R\right)}$, ${∥\mathrm{div}\tilde{b}∥}_{L^{\infty }\left(R^{d}\right)}$, $∥\tilde{b}{∥}_{L^{\infty }\left(R^{d}\right)}$ and T, such that*

(13) $$\begin{array}{ccc} \underset{0⩽t⩽T}{\sup}E\int_{R^{d}}\left|\rho \left(t,x\right)-\tilde{\rho }\left(t,x\right)\right|dx & ⩽ & \int_{R^{d}}\left|\rho_{0}\left(x\right)-{\tilde{\rho }}_{0}\left(x\right)\right|dx\\ & & +C[∥b-\tilde{b}{∥}_{L^{\infty }\left(R^{d}\right)}+∥f^{\prime }-{\tilde{f}}^{\prime }{∥}_{L^{\infty }\left(R\right)}]∥\rho_{0}{∥}_{BV(R^{d})}. \end{array}$$

**Remark** **3.** Without the noise, (1) has been discussed by Chen and Karlsen. Some results on the existence and uniqueness of solutions as well as continuous dependence on b and f have been obtained in [33]. Here, we get an analogue of [33] (Theorem 3.2) but simplify some assumptions on the velocity fields b and $\tilde{b}$.

The present paper is organized as follows. In Section 2, we give the proof of Theorem 1. Section 3 is devoted to the proof for Theorem 2. In Section 4, we prove the continuous dependence of solutions on *b* and *f*.

We end up this section by introducing some notations. N is natural numbers set. m∈N and $C_{0}^{m}\left(R^{d}\right)$ stands for the vector space consisting of all functions ϕ, which, together with all their partial derivatives $\partial^{\alpha }\phi$ of order |α|⩽m, are continuous and have compact supports in $R^{d}$. Given a measurable function ς, $\varsigma^{+}=\max\left\{\varsigma ,0\right\}=\varsigma \vee 0$ and $\varsigma^{-}=-\min\left\{\varsigma ,0\right\}=-\left[\varsigma \wedge 0\right]$. The symbols ∇, div, Δ, if not differently specified, are referred to derivatives in *x*. For every R>0, $B_{R}:=\{x\in R^{d}:|x|<R\}$. It almost surely can be abbreviated to a.s.. The letter *C* will mean a positive constant, whose values may change in different places.

## 2. Proof of Theorem 1

Let $\rho_{1}$ be a stochastic entropy solution of (1) with the initial data $\rho_{0}^{1}$ and $\rho_{2}$ be a stochastic strong entropy solution of (1) with the initial data $\rho_{0}^{2}$, respectively. We set $\rho_{12}\left(t,x\right):=\rho_{1}\left(t,x\right)-\rho_{2}\left(t,x\right)$ for every t>0 and $x\in R^{d}$.

Let ϱ be a 1-dimensional standard mollifier,

(14) $$\begin{array}{c} \varrho \left(r\right)=C_{0}\exp\left(\frac{1}{r^{2}-1}\right)1_{|r|<1}\left(r\right),\int_{R}\varrho \left(r\right)dr=1. \end{array}$$

For θ>0, we set $\varrho_{\theta }\left(r\right)=\varrho \left(r/\theta \right)/\theta$ and define

(15) $$\begin{array}{c} \eta_{\theta }\left(r\right)=\int_{-\infty }^{r}\int_{-\infty }^{s-\theta }\varrho_{\theta }\left(\tau \right)d\tau ds. \end{array}$$

For any δ>0 and any $0⩽\varphi \in C_{0}^{2}\left(R^{d}\right)$, we set

(16) $$\begin{array}{c} \psi_{\delta }\left(x,y\right)=\left(\delta^{-d}\prod_{k=1}^{d}\varrho \left(\frac{x_{k}-y_{k}}{\delta }\right)\right)\varphi \left(\frac{x+y}{2}\right)\in C_{0}^{2}\left(R^{2d}\right). \end{array}$$

If one chooses the entropy function by $\eta_{\theta }$, the test function by $\psi_{\delta }\left(x,y\right)$, and $\delta =\theta^{2/3}$, in view of the assumption $b\in BV_{loc}\left(R^{d};R^{d}\right)$, then all calculations from [11] (Lemma 3.1) to [11] (Lemma 3.3) are adapted to the present case. Furthermore, noting the fact that if $g\in L^{1}\left(R^{d}\right)$, for every ε>0, we define $g_{\varepsilon }\left(x\right)=1_{|g|⩽\varepsilon }\left(x\right)\left|g\left(x\right)\right|/\varepsilon$, then for almost everywhere $x\in R^{d}$,

(17) $$\begin{array}{c} g_{\varepsilon }\left(x\right)\to 0\;\mathrm{as}\;\;\varepsilon ↓0. \end{array}$$

Hence, Ref. [11] (Lemma 3.4) holds true as well if $A\in C^{\frac{1}{2}}\left(R\right)$.

Therefore, for every t>0, we conclude that

(18) $$\begin{array}{cc} & E\int_{R^{d}}\varphi \left(x\right){\left[\rho_{12}\right]}_{+}\left(t,x\right)dx-\int_{R^{d}}\varphi \left(x\right){\left[\rho_{12}\right]}_{+}\left(0,x\right)dx\\ ⩽ & E\int_{0}^{t}\int_{R^{d}}\left[\mathrm{div}b\left(x\right)\varphi \left(x\right)+b\left(x\right)\cdot \nabla \varphi \left(x\right)\right]1_{\left[0,\infty \right)}\left(\rho_{12}\left(r,x\right)\right)\left[f\left(\rho_{1}\left(r,x\right)\right)-f\left(\rho_{2}\left(r,x\right)\right)\right]dxdr\\ ⩽ & {∥f^{\prime }∥}_{L^{\infty }\left(\left[-a,a\right]\right)}E\int_{0}^{t}\int_{R^{d}}\left|\mathrm{div}b\left(x\right)\varphi \left(x\right)+b\left(x\right)\cdot \nabla \varphi \left(x\right)\right|{\left[\rho_{12}\right]}_{+}\left(r,x\right)dxdr, \end{array}$$

where $a=[\sup_{0⩽t⩽T}∥\rho_{1}{\left(t\right)∥}_{L^{\infty }\left(\Omega \times R^{d}\right)}]\vee [\sup_{0⩽t⩽T}∥\rho_{2}\left(t\right){∥}_{L^{\infty }\left(\Omega \times R^{d}\right)}]$.

Let the test function φ in (16) satisfy that $supp\left(\varphi \right)\subset B_{2}$, φ=1 on |x|⩽1. Let R>0 be a real number, and set $\varphi_{R}=\varphi \left(\cdot /R\right)$. With the help of (9): $\mathrm{div}b,\;|b\left(\cdot \right)|/(1+|\cdot |)\in L^{\infty }\left(R^{d}\right)$, if one takes φ(·/R) instead of φ in (18) and lets *R* tend to infinity, then

(19) $$\begin{array}{cc} & E\int_{R^{d}}{\left[\rho_{12}\right]}_{+}\left(t,x\right)dx-\int_{R^{d}}{\left[\rho_{12}\right]}_{+}\left(0,x\right)dx\\ ⩽ & {∥f^{\prime }∥}_{L^{\infty }\left(\left[-a,a\right]\right)}{∥\mathrm{div}b∥}_{L^{\infty }\left(R^{d}\right)}E\int_{0}^{t}\int_{R^{d}}{\left[\rho_{12}\right]}_{+}\left(r,x\right)dxdr. \end{array}$$

Thus, by the Grönwall inequality, one easily finds that

(20) $$\begin{array}{c} \underset{0⩽t⩽T}{\sup}E\int_{R^{d}}{\left[\rho_{12}\right]}_{+}\left(t,x\right)dx⩽\exp\{∥f^{\prime }{∥}_{L^{\infty }\left(\left[-a,a\right]\right)}{∥\mathrm{div}b∥}_{L^{\infty }\left(R^{d}\right)}T\}\int_{R^{d}}{\left[\rho_{12}\right]}_{+}\left(0,x\right)dx. \end{array}$$

Similar arguments imply that

(21) $$\begin{array}{c} \underset{0⩽t⩽T}{\sup}E\int_{R^{d}}{\left[\rho_{21}\right]}_{+}\left(t,x\right)dx⩽\exp\{∥f^{\prime }{∥}_{L^{\infty }\left(\left[-a,a\right]\right)}{∥\mathrm{div}b∥}_{L^{\infty }\left(R^{d}\right)}T\}\int_{R^{d}}{\left[\rho_{21}\right]}_{+}\left(0,x\right)dx. \end{array}$$

Combining (20) and (21), we complete the proof. □

## 3. Proof of Theorem 2

(i) We prove the existence of stochastic strong entropy solutions for (1) by the method of vanishing viscosity, that is, we regard (1) as the ε↓0 limit of the viscosity equation

(22) $$\begin{array}{c} \left\{\begin{array}{c} d\rho^{\varepsilon }\left(t,x\right)+b\left(x\right)\cdot \nabla f\left(\rho^{\varepsilon }\left(t,x\right)\right)dt=\varepsilon \Delta \rho^{\varepsilon }\left(t,x\right)dt+A\left(\rho^{\varepsilon }\left(t,x\right)\right)dW_{t},\;t>0,\;x\in R^{d},\\ \rho^{\varepsilon }{\left(t,x\right)|}_{t=0}=\rho_{0}^{\varepsilon }\left(x\right),\;x\in R^{d}, \end{array}\right. \end{array}$$

where $\rho_{0}^{\varepsilon }$ is an approximation to $\rho_{0}$.

We now divide the proof into three steps. 

**Step 1.** Existence and uniqueness of mild solutions to the Cauchy problem (22). 

Here, $\rho^{\varepsilon }$ is said to be a mild solution of (22), if $\rho^{\varepsilon }\left(t\right)$ is an $F_{t}$-adapted $L^{2}\left(R^{d}\right)$-valued stochastic process and satisfies

(23) $$\begin{array}{ccc} \rho^{\varepsilon }\left(t,x\right) & = & \int_{R^{d}}G_{\varepsilon }\left(t,x-z\right)\rho_{0}^{\varepsilon }\left(z\right)dz+\int_{0}^{t}\int_{R^{d}}{\mathrm{div}}_{z}\left(G_{\varepsilon }\left(t-r,x-z\right)b\left(z\right)\right)f\left(\rho^{\varepsilon }\left(r,z\right)\right)dzdr\\ & & +\int_{0}^{t}dW_{r}\int_{R^{d}}G_{\varepsilon }\left(t-r,x-z\right)A\left(\rho^{\varepsilon }\left(r,z\right)\right)dz,\;P-a.s., \end{array}$$

for every t⩾0, almost everywhere $x\in R^{d}$, where the heat kernel $G_{\varepsilon }\left(t,x\right)=e^{-\frac{{\left|x\right|}^{2}}{4\varepsilon t}}/{\left(4\pi \varepsilon t\right)}^{d/2}.$

We choose $\rho_{0}^{\varepsilon }\in L^{1}\cap L^{\infty }\cap H^{1}\left(R^{d}\right)$ such that $\rho_{0}^{\varepsilon }\to \rho_{0}$ in $L^{1}\cap L^{2}\left(R^{d}\right)$ as ε↓0. For every fixed ε, every p∈[1,∞], ${∥\rho_{0}^{\varepsilon }∥}_{L^{p}\left(R^{d}\right)}⩽{∥\rho_{0}∥}_{L^{p}\left(R^{d}\right)}$. With the help of Banach contraction mapping principle, there is a unique mild solution $\rho^{\varepsilon }$ to (22). Moreover, for every T>0,

$$\rho^{\varepsilon }\in C\left(\left[0,T\right];L^{2}\left(\Omega ;H^{1}\left(R^{d}\right)\right)\right)\cap L^{2}\left(\left[0,T\right]\times \Omega ;H^{2}\left(R^{d}\right)\right)\cap L^{\infty }\left(\left[0,T\right];L^{p}\left(\Omega \times R^{d}\right)\right),\;\forall p\in \left[1,\infty \right).$$

Furthermore, for every 1⩽p<∞, every T>0, we have

(24) $$\begin{array}{cc} & \underset{0⩽t⩽T}{\sup}E\left[{∥\rho^{\varepsilon }\left(t\right)∥}_{L^{p}\left(R^{d}\right)}^{p}\right]+E\left[\varepsilon \int_{0}^{T}{∥\nabla \rho^{\varepsilon }\left(t\right)∥}_{L^{2}\left(R^{d}\right)}^{2}dt\right]\\ ⩽ & C\left({∥b∥}_{L^{\infty }\left(R^{d}\right)},{∥\mathrm{div}b∥}_{L^{\infty }\left(R^{d}\right)},{∥f^{\prime }∥}_{L^{\infty }\left(R\right)},T\right)\left[{∥\rho_{0}^{\varepsilon }∥}_{L^{p}\left(R^{d}\right)}^{p}+{∥\rho_{0}^{\varepsilon }∥}_{L^{2}\left(R^{d}\right)}^{2}\right]\\ ⩽ & C\left({∥b∥}_{L^{\infty }\left(R^{d}\right)},{∥\mathrm{div}b∥}_{L^{\infty }\left(R^{d}\right)},{∥f^{\prime }∥}_{L^{\infty }\left(R\right)},T\right)\left[{∥\rho_{0}∥}_{L^{p}\left(R^{d}\right)}^{p}+{∥\rho_{0}∥}_{L^{2}\left(R^{d}\right)}^{2}\right] \end{array}$$

and

(25) $$\begin{array}{c} E∥\nabla^{2}\rho^{\varepsilon }{∥}_{L^{2}\left(\left[0,T\right]\times R^{d}\right)}⩽{C(∥b∥}_{W^{1,\infty }\left(R^{d}\right)},∥f^{\prime }{∥}_{L^{\infty }\left(R\right)},\varepsilon )∥\nabla \rho_{0}^{\varepsilon }{∥}_{L^{2}\left(R^{d}\right)}. \end{array}$$

We show that (4) holds for $\rho^{\varepsilon }$. Let $\eta_{\theta }$ be given by (15). ∀M>0, define $\eta_{\theta }^{M}\left(r\right)=\eta_{\theta }\left(r-M\right)$, then

(26) $$\begin{array}{c} \eta_{\theta }^{M}\left(r\right)\to {\left(r-M\right)}_{+}\;\;\mathrm{as}\;\;\theta ↓0. \end{array}$$

Let $\tilde{\varrho }$ be a *d*-dimensional standard mollifier, i.e.,

(27) $$\begin{array}{c} \tilde{\varrho }\left(x\right)=C_{1}\exp\left(\frac{1}{{\left|x\right|}^{2}-1}\right)1_{|x|<1}\left(x\right),\int_{R^{d}}\tilde{\varrho }\left(x\right)dx=1. \end{array}$$

For δ>0, we define ${\tilde{\varrho }}_{\delta }\left(x\right)=\tilde{\varrho }\left(x/\delta \right)/\delta^{d}$. Let $\varphi \left(x\right)=C_{1}e^{-|x|}$, with $C_{1}={\left[\int_{R^{d}}e^{-|x|}dx\right]}^{-1}$ and for every given natural number n∈N, we set $\varphi_{\delta }^{n}\left(x\right)=\left(\varphi 1_{|x|<n}\left(\cdot \right)\right)*{\tilde{\varrho }}_{\delta }\left(x\right)$.

By using Itô’s formula and the integration by parts, then

(28) $$\begin{array}{cc} & E\int_{R^{d}}\varphi_{\delta }^{n}\left(x\right)\eta_{\theta }^{M}\left(\rho^{\varepsilon }\left(t,x\right)\right)dx-\int_{R^{d}}\varphi_{\delta }^{n}\left(x\right)\eta_{\theta }^{M}\left(\rho_{0}^{\varepsilon }\left(x\right)\right)dx\\ ⩽ & E\int_{0}^{t}\int_{R^{d}}\mathrm{div}\left(b\left(x\right)\varphi_{\delta }^{n}\left(x\right)\right)q_{M}^{\delta }\left(\rho^{\varepsilon }\left(r,x\right)\right)dxdr+\varepsilon E\int_{0}^{t}\int_{R^{d}}\eta_{\theta }^{M}\left(\rho^{\varepsilon }\left(r,x\right)\right)\Delta \varphi_{\delta }^{n}\left(x\right)dxdr\\ & +\frac{1}{2}E\int_{0}^{t}\int_{R^{d}}{\left(\eta_{\theta }^{M}\right)}^{\prime \prime }\left(\rho^{\varepsilon }\left(r,x\right)\right)A^{2}\left(\rho^{\varepsilon }\left(r,x\right)\right)\varphi_{\delta }^{n}\left(x\right)dxdr, \end{array}$$

where in (28) we have used the fact

(29) $$\begin{array}{c} \Delta \eta_{\theta }^{M}\left(\rho^{\varepsilon_{1}}\left(t,x\right)\right)⩾{\left(\eta_{\theta }^{M}\right)}^{\prime }\left(\rho^{\varepsilon_{1}}\left(t,x\right)\right)\Delta \rho^{\varepsilon_{1}}\left(t,x\right). \end{array}$$

For θ,δ,M and ε be fixed, if one lets *n* approach to infinity, (28) turns to

$$\begin{array}{cc} & E\int_{R^{d}}\varphi_{\delta }\left(x\right)\eta_{\theta }^{M}\left(\rho^{\varepsilon }\left(t,x\right)\right)dx-\int_{R^{d}}\varphi_{\delta }\left(x\right)\eta_{\theta }^{M}\left(\rho_{0}^{\varepsilon }\left(x\right)\right)dx\\ ⩽ & E\int_{0}^{t}\int_{R^{d}}\mathrm{div}\left(b\left(x\right)\varphi_{\delta }\left(x\right)\right)q_{\theta }^{M}\left(\rho^{\varepsilon }\left(r,x\right)\right)dxdr+\varepsilon E\int_{0}^{t}\int_{R^{d}}\eta_{\theta }^{M}\left(\rho^{\varepsilon }\left(r,x\right)\right)\Delta \varphi_{\delta }\left(x\right)dxdr\\ & +\frac{1}{2}E\int_{0}^{t}\int_{R^{d}}{\left(\eta_{\theta }^{M}\right)}^{\prime \prime }\left(\rho^{\varepsilon }\left(r,x\right)\right)A^{2}\left(\rho^{\varepsilon }\left(r,x\right)\right)\varphi_{\delta }\left(x\right)dxdr\\ ⩽ & E\int_{0}^{t}\int_{R^{d}}\mathrm{div}\left(b\left(x\right)\varphi_{\delta }\left(x\right)\right)q_{\theta }^{M}\left(\rho^{\varepsilon }\left(r,x\right)\right)dxdr+\varepsilon E\int_{0}^{t}\int_{R^{d}}\eta_{\theta }^{M}\left(\rho^{\varepsilon }\left(r,x\right)\right)\varphi_{\delta }\left(x\right)dxdr\\ & +CE\int_{0}^{t}\int_{R^{d}}\frac{1}{\theta }1_{|\rho^{\varepsilon }\left(r,x\right)-M|⩽\theta }A^{2}\left(\rho^{\varepsilon }\left(r,x\right)\right)\varphi_{\delta }\left(x\right)dxdr, \end{array}$$

where $\varphi_{\delta }\left(x\right)=\left(\varphi *{\tilde{\varrho }}_{\delta }\right)\left(x\right)$ and in the last inequality we use the fact $\Delta \varphi_{\delta }\left(x\right)⩽\varphi_{\delta }\left(x\right)$. Then, taking δ→0, we arrive at

(30) $$\begin{array}{cc} & E\int_{R^{d}}\varphi \left(x\right)\eta_{\theta }^{M}\left(\rho^{\varepsilon }\left(t,x\right)\right)dx-\int_{R^{d}}\varphi \left(x\right)\eta_{\theta }^{M}\left(\rho_{0}^{\varepsilon }\left(x\right)\right)dx\\ ⩽ & E\int_{0}^{t}\int_{R^{d}}\mathrm{div}\left(b\left(x\right)\varphi \left(x\right)\right)q_{\theta }^{M}\left(\rho^{\varepsilon }\left(r,x\right)\right)dxdr+\varepsilon E\int_{0}^{t}\int_{R^{d}}\eta_{\theta }^{M}\left(\rho^{\varepsilon }\left(r,x\right)\right)\varphi \left(x\right)dxdr\\ & +CE\int_{0}^{t}\int_{R^{d}}\frac{1}{\theta }1_{|\rho^{\varepsilon }\left(r,x\right)-M|⩽\theta }A^{2}\left(\rho^{\varepsilon }\left(r,x\right)\right)\varphi \left(x\right)dxdr. \end{array}$$

Observing that $f^{\prime }$ is bounded, $\left(\eta_{\theta }^{M}\right)\left(M\right)={\left(\eta_{\theta }^{M}\right)}^{\prime }\left(M\right)=0$ and ${\left(\eta_{\theta }^{M}\right)}^{\prime \prime }⩾0$, then

(31) $$\begin{array}{c} \left|q_{\theta }^{M}\left(\rho^{\varepsilon }\right)\right|=\left|\int_{M}^{\rho^{\varepsilon }}f^{\prime }\left(v\right){\left(\eta_{\theta }^{M}\right)}^{\prime }\left(v\right)dv\right|⩽{∥f^{\prime }∥}_{L^{\infty }\left(R\right)}\left|\int_{M}^{\rho^{\varepsilon }}{\left(\eta_{\theta }^{M}\right)}^{\prime }\left(v\right)dv\right|={∥f^{\prime }∥}_{L^{\infty }\left(R\right)}\eta_{\theta }^{M}\left(\rho^{\varepsilon }\right). \end{array}$$

By virtue of (11), taking M>N, from (30) and (31), we have

$$\begin{array}{cc} & E\int_{R^{d}}\varphi \left(x\right)\eta_{\theta }^{M}\left(\rho^{\varepsilon }\left(t,x\right)\right)dx-\int_{R^{d}}\varphi \left(x\right)\eta_{\theta }^{M}\left(\rho_{0}^{\varepsilon }\left(x\right)\right)dx\\ ⩽ & {[C(∥b∥}_{W^{1,\infty }\left(R^{d}\right)},∥f^{\prime }{∥}_{L^{\infty }\left(R\right)},T)+\varepsilon ]E\int_{0}^{t}\int_{R^{d}}\varphi \left(x\right)\eta_{\theta }^{M}\left(\rho^{\varepsilon }\left(r,x\right)\right)dxdr+C\theta , \end{array}$$

for all 0⩽t⩽T (T>0 is a given real number). Therefore,

$$\begin{array}{c} E\int_{R^{d}}\varphi \left(x\right)\eta_{\theta }^{M}\left(\rho^{\varepsilon }\left(t,x\right)\right)dx⩽C\int_{R^{d}}\varphi \left(x\right)\eta_{\theta }^{M}\left(\rho_{0}^{\varepsilon }\left(x\right)\right)dx+C\theta , \end{array}$$

uniformly for ε⩽1.

Due to (26), letting θ↓0, for $M>∥\rho_{0}^{\varepsilon }{∥}_{L^{\infty }\left(R^{d}\right)}$, we get

(32) $$\begin{array}{c} E\int_{R^{d}}\varphi \left(x\right){\left(\rho^{\varepsilon }\left(t,x\right)-M\right)}_{+}dx⩽C\int_{R^{d}}\varphi \left(x\right){\left(\rho_{0}^{\varepsilon }\left(x\right)-M\right)}_{+}dx=0. \end{array}$$

Since $\rho^{\varepsilon }$ is in $C(\left[0,T\right];L^{2}\left(\Omega \times R^{d}\right))$, using the dominated convergence theorem, for $M>∥\rho_{0}^{\varepsilon }{∥}_{L^{\infty }\left(R^{d}\right)}$, from (32), one has

(33) $$\begin{array}{c} E\int_{R^{d}}\varphi \left(x\right){\left(\rho^{\varepsilon }\left(t,x\right)-M\right)}_{+}^{2}dx=0,\;\forall \;t\in \left[0,T\right]. \end{array}$$

By the convexity of $\eta_{\theta }^{M}$, with the help of (28), (32) and (33), if $M>\max\{N,∥\rho_{0}^{\varepsilon }{∥}_{L^{\infty }\left(R^{d}\right)}\}$, we have

$$\begin{array}{cc} & E\underset{0⩽t⩽T}{\sup}\int_{R^{d}}\varphi \left(x\right){\left(\rho^{\varepsilon }\left(t,x\right)-M\right)}_{+}dx\\ ⩽ & C\int_{R^{d}}\varphi \left(x\right){\left(\rho_{0}^{\varepsilon }\left(x\right)-M\right)}_{+}dx+CE\int_{0}^{T}\int_{R^{d}}{\left(\rho^{\varepsilon }\left(t,x\right)-M\right)}_{+}\varphi \left(x\right)dxdt\\ & +C{\left[E\int_{0}^{T}|\int_{R^{d}}{\left(\rho^{\varepsilon }\left(t,x\right)-M\right)}_{+}\varphi \left(x\right)dx{|}^{2}dt\right]}^{\frac{1}{2}}\\ ⩽ & C\int_{R^{d}}\varphi \left(x\right){\left(\rho_{0}^{\varepsilon }\left(x\right)-M\right)}_{+}dx+CE\int_{0}^{T}\int_{R^{d}}{\left(\rho^{\varepsilon }\left(t,x\right)-M\right)}_{+}\varphi \left(x\right)dxdt\\ & +C{\left[E\int_{0}^{T}\int_{R^{d}}{\left(\rho^{\varepsilon }\left(t,x\right)-M\right)}_{+}^{2}\varphi \left(x\right)dxdt\right]}^{\frac{1}{2}}=0. \end{array}$$

For the above calculations for $\eta_{\theta }^{M}$ adapted to $\xi_{\theta }^{M}=\xi_{\theta }\left(r+M\right)$, if $M>\max\{N,∥\rho_{0}^{\varepsilon }{∥}_{L^{\infty }\left(R^{d}\right)}\}$, we have

$$\begin{array}{c} E\underset{0⩽t⩽T}{\sup}\int_{R^{d}}\varphi \left(x\right){\left(\rho^{\varepsilon }\left(t,x\right)+M\right)}_{-}dx⩽C\int_{R^{d}}\varphi \left(x\right){\left(\rho_{0}^{\varepsilon }\left(x\right)+M\right)}_{-}dx=0, \end{array}$$

where $\xi_{\theta }\left(r\right)=\xi \left(r/\theta \right)/\theta$, ξ:R→R is a $C^{\infty }$ convex function satisfying

$$\xi \left(0\right)=0,\;\;\xi^{\prime }\left(r\right)\left\{\begin{array}{cc} =0, & \mathrm{when}\;r>0,\\ \in [-1,0], & \mathrm{when}\;-2⩽r⩽0,\\ =-1, & \mathrm{when}\;r<-2. \end{array}\right.$$

Therefore, (4) is true for $\rho^{\varepsilon }$, and

(34) $$\begin{array}{c} \underset{0⩽t⩽T}{\sup}∥\rho^{\varepsilon }{\left(t\right)∥}_{L^{\infty }\left(\Omega \times R^{d}\right)}⩽\max\{N,∥\rho_{0}{∥}_{L^{\infty }\left(R^{d}\right)}\}. \end{array}$$

**Step 2.** Existence of the stochastic entropy solution to the Cauchy problem (1).

We choose $\rho_{0}^{\varepsilon }$ as in **Step 1**, and when $\varepsilon =\varepsilon_{i}\;\left(i=1,2\right)$ in (22), we use the notation $\rho^{\varepsilon_{i}}\;\left(i=1,2\right)$ to denote the unique stochastic entropy solution now. Suppose that $\eta_{\theta }$ is given by (15), then

(35) $$\begin{array}{c} \Delta_{y}\eta_{\theta }\left(\rho^{\varepsilon_{1}}\left(t,x\right)-\rho^{\varepsilon_{2}}\left(t,y\right)\right)⩾-\eta_{\theta }^{\prime }\left(\rho^{\varepsilon_{1}}\left(t,x\right)-\rho^{\varepsilon_{2}}\left(t,y\right)\right)\Delta_{y}\rho^{\varepsilon_{2}}\left(t,y\right). \end{array}$$

Let $0⩽J,\varphi \in C_{0}^{2}\left(R^{d}\right)$, such that

(36) $$\begin{array}{c} \left\{\begin{array}{cc} J(x)=0, & \mathrm{when}\;|x|⩾1,\\ |\nabla_{x}J\left(x\right)|⩽CJ\left(x\right), & \mathrm{when}\;x\in R^{d},\\ \int_{R^{d}}J\left(x\right)dx=1. \end{array}\right.\;\left\{\begin{array}{cc} \;\varphi (x)=1, & \mathrm{when}\;|x|⩽1,\\ |\nabla_{x}\varphi \left(x\right)|⩽C\varphi \left(x\right), & \mathrm{when}\;x\in R^{d}. \end{array}\right. \end{array}$$

For any δ>0, we set

$$\begin{array}{c} \psi_{\delta }\left(x,y\right)=J_{\delta }\left(x-y\right)\varphi \left(\frac{x+y}{2}\right)=\delta^{-d}J\left(\frac{x-y}{\delta }\right)\varphi \left(\frac{x+y}{2}\right)\in C_{0}^{2}\left(R^{2d}\right). \end{array}$$

In view of (29) and (35), by using Itô’s formula and the integration by parts,

(37) $$\begin{array}{cc} & \int_{R^{2d}}\psi_{\delta }\left(x,y\right)\eta_{\theta }\left(\rho^{\varepsilon_{1}}\left(t,x\right)-\rho^{\varepsilon_{2}}\left(t,y\right)\right)dxdy-\int_{R^{2d}}\psi_{\delta }\left(x,y\right)\eta_{\theta }\left(\rho_{0}^{\varepsilon_{1}}\left(x\right)-\rho_{0}^{\varepsilon_{2}}\left(y\right)\right)dxdy\\ ⩽ & \int_{0}^{t}\int_{R^{2d}}\left[{\mathrm{div}}_{x}\left(b\left(x\right)\psi_{\delta }\right)q_{\theta }^{\varepsilon_{1}}\left(\rho^{\varepsilon_{1}}\left(r,x\right),\rho^{\varepsilon_{2}}\left(r,y\right)\right)+{\mathrm{div}}_{y}\left(b\left(y\right)\psi_{\delta }\right){\hat{q}}_{\theta }^{\varepsilon_{2}}\left(\rho^{\varepsilon_{1}}\left(r,x\right),\rho^{\varepsilon_{2}}\left(r,y\right)\right)\right]dxdydr\\ & +\frac{1}{2}\int_{0}^{t}\int_{R^{2d}}\psi_{\delta }\left(x,y\right)\eta_{\theta }^{\prime \prime }\left(\rho^{\varepsilon_{1}}\left(r,x\right)-\rho^{\varepsilon_{2}}\left(r,y\right)\right){\left|A\left(\rho^{\varepsilon_{1}}\left(r,x\right)\right)-A\left(\rho^{\varepsilon_{2}}\left(r,y\right)\right)\right|}^{2}dxdydr\\ & +\int_{0}^{t}\int_{R^{2d}}\eta_{\theta }\left(\rho^{\varepsilon_{1}}\left(r,x\right)-\rho^{\varepsilon_{2}}\left(r,y\right)\right)\left[\varepsilon_{1}\Delta_{x}+\varepsilon_{2}\Delta_{y}\right]\psi_{\delta }\left(x,y\right)dxdydr\\ & +\int_{0}^{t}dW_{r}\int_{R^{2d}}\psi_{\delta }\left(x,y\right)\eta^{\prime }\left(\rho^{\varepsilon_{1}}\left(r,x\right)-\rho^{\varepsilon_{2}}\left(r,y\right)\right)\left[A\left(\rho^{\varepsilon_{1}}\left(r,x\right)\right)-A\left(\rho^{\varepsilon_{2}}\left(r,y\right)\right)\right]dxdy\\ = & :H_{1}\left(t\right)+H_{2}\left(t\right)+H_{3}\left(t\right)+H_{4}\left(t\right), \end{array}$$

where

$$\begin{array}{ccc} q_{\theta }^{\varepsilon_{1}}\left(\rho^{\varepsilon_{1}}\left(r,x\right),\rho^{\varepsilon_{2}}\left(r,y\right)\right) & = & \int_{\rho^{\varepsilon_{2}}\left(r,y\right)}^{\rho^{\varepsilon_{1}}\left(r,x\right)}\eta_{\theta }^{\prime }\left(v-\rho^{\varepsilon_{2}}\left(r,y\right)\right)f^{\prime }\left(v\right)dv,\\ {\hat{q}}_{\theta }^{\varepsilon_{2}}\left(\rho^{\varepsilon_{1}}\left(r,x\right),\rho^{\varepsilon_{2}}\left(r,y\right)\right) & = & \int_{\rho^{\varepsilon_{2}}\left(r,y\right)}^{\rho^{\varepsilon_{1}}\left(r,x\right)}\eta_{\theta }^{\prime }\left(\rho^{\varepsilon_{1}}\left(r,x\right)-v\right)f^{\prime }\left(v\right)dv. \end{array}$$

Clearly, $EH_{4}\left(t\right)=0$. For $\varepsilon_{1},\varepsilon_{2}$ and δ are fixed, then

(38) $$\begin{array}{ccc} \lim_{\theta ↓0}EH_{1}\left(t\right) & = & \int_{0}^{t}\int_{R^{2d}}\left[{\mathrm{div}}_{x}\left(b\left(x\right)\psi_{\delta }\right)+{\mathrm{div}}_{y}\left(b\left(y\right)\psi_{\delta }\right)\right]1_{\left[0,\infty \right)}\left(\rho^{\varepsilon_{1}}\left(r,x\right)-\rho^{\varepsilon_{2}}\left(r,y\right)\right)\\ & & \times [f\left(\rho^{\varepsilon_{1}}\left(r,x\right)\right)-f\left(\rho^{\varepsilon_{2}}\left(r,y\right)\right)]dxdydr\\ & ⩽ & C\int_{0}^{t}\int_{R^{2d}}\left|{\mathrm{div}}_{x}\left(b\left(x\right)\psi_{\delta }\right)+{\mathrm{div}}_{y}\left(b\left(y\right)\psi_{\delta }\right)\right|{\left[\rho^{\varepsilon_{1}}\left(r,x\right)-\rho^{\varepsilon_{2}}\left(r,y\right)\right]}_{+}dxdydr\\ & ⩽ & {C∥\mathrm{div}b∥}_{L^{\infty }\left(R^{d}\right)}\int_{0}^{t}\int_{R^{2d}}\psi_{\delta }\left(x,y\right){\left[\rho^{\varepsilon_{1}}\left(r,x\right)-\rho^{\varepsilon_{2}}\left(r,y\right)\right]}_{+}dxdydr\\ & & +{C∥\nabla b∥}_{L^{\infty }\left(R^{d}\right)}\int_{0}^{t}\int_{R^{2d}}\varphi \left(\frac{x+y}{2}\right)\left|\nabla_{x}J_{\delta }\left(x-y\right)\right|{\left[\rho^{\varepsilon_{1}}\left(r,x\right)-\rho^{\varepsilon_{2}}\left(r,y\right)\right]}_{+}dxdydr\\ & & +{C∥b∥}_{L^{\infty }\left(R^{d}\right)}\int_{0}^{t}\int_{R^{2d}}\left|\nabla_{x}\varphi \left(\frac{x+y}{2}\right)\right|J_{\delta }\left(x-y\right){\left[\rho^{\varepsilon_{1}}\left(r,x\right)-\rho^{\varepsilon_{2}}\left(r,y\right)\right]}_{+}dxdydr.\\ & ⩽ & C\int_{0}^{t}\int_{R^{2d}}\psi_{\delta }\left(x,y\right){\left[\rho^{\varepsilon_{1}}\left(r,x\right)-\rho^{\varepsilon_{2}}\left(r,y\right)\right]}_{+}dxdydr, \end{array}$$

where in the last inequality we have used (36).

Moreover, $\lim_{\theta ↓0}EH_{2}\left(t\right)=0$ and

(39) $$\begin{array}{c} \lim_{\theta ↓0}EH_{3}\left(t\right)=\int_{0}^{t}\int_{R^{2d}}{\left[\rho^{\varepsilon_{1}}\left(r,x\right)-\rho^{\varepsilon_{2}}\left(r,y\right)\right]}_{+}\left[\varepsilon_{1}\Delta_{x}+\varepsilon_{2}\Delta_{y}\right]\psi_{\delta }\left(x,y\right)dxdydr. \end{array}$$

For every T>0, by (37)–(39), we obtain

(40) $$\begin{array}{cc} & \underset{0⩽t⩽T}{\sup}\left[E\int_{R^{2d}}\psi_{\delta }\left(x,y\right){\left[\rho^{\varepsilon_{1}}\left(t,x\right)-\rho^{\varepsilon_{2}}\left(t,y\right)\right]}_{+}dxdy\right]-\int_{R^{2d}}\psi_{\delta }\left(x,y\right){\left[\rho_{0}^{\varepsilon_{1}}\left(x\right)-\rho_{0}^{\varepsilon_{2}}\left(y\right)\right]}_{+}dxdy\\ ⩽ & \underset{0⩽t⩽T}{\sup}C\left[E\int_{0}^{t}\int_{R^{2d}}\psi_{\delta }\left(x,y\right){\left[\rho^{\varepsilon_{1}}\left(r,x\right)-\rho^{\varepsilon_{2}}\left(r,y\right)\right]}_{+}dxdydr\right]\\ & +\underset{0⩽t⩽T}{\sup}\left[E\int_{0}^{t}\int_{R^{2d}}{\left[\rho^{\varepsilon_{1}}\left(r,x\right)-\rho^{\varepsilon_{2}}\left(r,y\right)\right]}_{+}\left[\varepsilon_{1}\Delta_{x}+\varepsilon_{2}\Delta_{y}\right]\psi_{\delta }\left(x,y\right)dxdydr\right]. \end{array}$$

Observing that

$$\begin{array}{c} \left|\left[\varepsilon_{1}\Delta_{x}+\varepsilon_{2}\Delta_{y}\right]\psi_{\delta }\left(x,y\right)\right|⩽C\frac{\varepsilon_{1}+\varepsilon_{2}}{\delta^{2}}{\tilde{\psi }}_{\delta }\left(x,y\right), \end{array}$$

where

$$\begin{array}{c} {\tilde{\psi }}_{\delta }\left(x,y\right)=\frac{1}{\delta^{d}}\tilde{J}\left(\frac{x-y}{\delta }\right)\tilde{\varphi }\left(\frac{x+y}{2}\right)\in C_{0}\left(R^{2d}\right),\;\;\tilde{J},\tilde{\varphi }\in C_{0}\left(R^{d}\right). \end{array}$$

With the help of dominated convergence theorem, then

(41) $$\begin{array}{c} \lim_{\varepsilon_{1}↓0,\varepsilon_{2}↓0,\delta ↓0,\frac{\varepsilon_{1}+\varepsilon_{2}}{\delta^{2}}\to 0}\underset{0⩽t⩽T}{\sup}\lim_{\theta ↓0}EH_{3}\left(t\right)=0. \end{array}$$

Combining (40), (41), and with the aid of Grönwall’s inequality, then

$$\begin{array}{c} \lim_{\varepsilon_{1}↓0,\varepsilon_{2}↓0,\delta ↓0,\frac{\varepsilon_{1}+\varepsilon_{2}}{\delta^{2}}\to 0}\underset{0⩽t⩽T}{\sup}E\int_{R^{2d}}\psi_{\delta }\left(x,y\right){\left[\rho^{\varepsilon_{1}}\left(t,x\right)-\rho^{\varepsilon_{2}}\left(t,y\right)\right]}_{+}dxdy=0. \end{array}$$

Similar arguments also hint that

$$\begin{array}{c} \lim_{\varepsilon_{1}↓0,\varepsilon_{2}↓0,\delta ↓0,\frac{\varepsilon_{1}+\varepsilon_{2}}{\delta^{2}}\to 0}\underset{0⩽t⩽T}{\sup}E\int_{R^{2d}}\psi_{\delta }\left(x,y\right){\left[\rho^{\varepsilon_{1}}\left(t,x\right)-\rho^{\varepsilon_{2}}\left(t,y\right)\right]}_{-}dxdy=0. \end{array}$$

Therefore,

(42) $$\begin{array}{c} \lim_{\varepsilon_{1}↓0,\varepsilon_{2}↓0,\delta ↓0,\frac{\varepsilon_{1}+\varepsilon_{2}}{\delta^{2}}\to 0}\underset{0⩽t⩽T}{\sup}E\int_{R^{2d}}\psi_{\delta }\left(x,y\right)\left|\rho^{\varepsilon_{1}}\left(t,x\right)-\rho^{\varepsilon_{2}}\left(t,y\right)\right|dxdy=0. \end{array}$$

On the other hand, we have

(43) $$\begin{array}{cc} & \int_{R^{2d}}\psi_{\delta }\left(x,y\right)\left|\rho^{\varepsilon_{1}}\left(t,x\right)-\rho^{\varepsilon_{2}}\left(t,y\right)\right|dxdy\\ = & \int_{R^{2d}}J\left(u\right)\varphi \left(v\right)\left|\rho^{\varepsilon_{1}}\left(t,v+\frac{\delta u}{2}\right)-\rho^{\varepsilon_{2}}\left(t,v-\frac{\delta u}{2}\right)\right|dudv\\ = & \int_{R^{2d}}J\left(u\right)\varphi \left(v\right)\left|\rho^{\varepsilon_{1}}\left(t,v\right)-\rho^{\varepsilon_{2}}\left(t,v-\delta u\right)\right|dudv\\ & +\int_{R^{2d}}J\left(u\right)\left[\varphi \left(v-\delta u\right)-\varphi \left(v\right)\right]\left|\rho^{\varepsilon_{1}}\left(t,v\right)-\rho^{\varepsilon_{2}}\left(t,v-\delta u\right)\right|dudv. \end{array}$$

In view of (34),

(44) $$\begin{array}{c} \underset{\delta ↓0}{lim sup}\underset{\varepsilon_{1},\varepsilon_{2}}{\sup}\underset{0⩽t⩽T}{\sup}E\int_{R^{2d}}J\left(u\right)|\varphi \left(v-\delta u\right)-\varphi \left(v\right)||\rho^{\varepsilon_{1}}\left(t,v\right)-\rho^{\varepsilon_{2}}\left(t,v-\delta u\right)|dudv=0. \end{array}$$

By (42)–(44), then

(45) $$\begin{array}{c} \lim_{\varepsilon_{1}↓0,\varepsilon_{2}↓0,\delta ↓0,\frac{\varepsilon_{1}+\varepsilon_{2}}{\delta^{2}}\to 0}\underset{0⩽t⩽T}{\sup}E\int_{R^{2d}}J\left(u\right)\varphi \left(v\right)\left|\rho^{\varepsilon_{1}}\left(t,v\right)-\rho^{\varepsilon_{2}}\left(t,v-\delta u\right)\right|dudv=0. \end{array}$$

Let *J* and φ be given in (36), then, for $\delta ={\left(\varepsilon_{1}\wedge \varepsilon_{2}\right)}^{1/3},$ we have

$$\begin{array}{cc} & \int_{R^{d}}\varphi \left(v\right)|\rho^{\varepsilon_{1}}\left(t,v\right)-\rho^{\varepsilon_{2}}\left(t,v\right)|dv=\int_{R^{2d}}J\left(u\right)\varphi \left(v\right)\left|\rho^{\varepsilon_{1}}\left(t,v\right)-\rho^{\varepsilon_{2}}\left(t,v\right)\right|dvdu\\ ⩽ & \int_{R^{2d}}J\left(u\right)\varphi \left(v\right)|\rho^{\varepsilon_{1}}\left(t,v\right)-\rho^{\varepsilon_{2}}\left(t,v-\delta u\right)|dvdu+\int_{R^{2d}}J\left(u\right)\varphi \left(v\right)\left|\rho^{\varepsilon_{2}}\left(t,v\right)-\rho^{\varepsilon_{2}}\left(t,v-\delta u\right)\right|dvdu. \end{array}$$

We conclude that

(46) $$\begin{array}{c} \lim_{\varepsilon_{1}↓0,\varepsilon_{2}↓0}\underset{0⩽t⩽T}{\sup}E\int_{R^{d}}\varphi \left(v\right)\left|\rho^{\varepsilon_{1}}\left(t,v\right)-\rho^{\varepsilon_{2}}\left(t,v\right)\right|dv=0. \end{array}$$

Let R>0 be a real number. If one takes $\varphi_{R}\left(x\right)=\varphi \left(x/R\right)$ instead of φ in the above calculations, then we get an analogue of (46),

(47) $$\begin{array}{c} \lim_{\varepsilon_{1}↓0,\varepsilon_{2}↓0}\underset{0⩽t⩽T}{\sup}E\int_{R^{d}}\varphi_{R}\left(v\right)\left|\rho^{\varepsilon_{1}}\left(t,v\right)-\rho^{\varepsilon_{2}}\left(t,v\right)\right|dv=0. \end{array}$$

Thus, there is an $F_{t}$-adapted $L_{loc}^{1}$ valued random process ρ(t), such that: $\rho \in C(\left[0,T\right];L^{1}\left(\Omega ;L_{loc}^{1}\left(R^{d}\right)\right))$ and $\rho^{\varepsilon }\to \rho$ in $C(\left[0,T\right];L^{1}\left(\Omega ;L_{loc}^{1}\left(R^{d}\right)\right))$. Moreover, by applying the estimates (24) and (34), (4) holds true.

On the other hand, for every entropy flux pair (η,q) ($\eta \in C^{\infty }\left(R\right)$, $\eta^{\prime \prime }⩾0$ and $q\left(v\right)=\int^{v}f^{\prime }\left(s\right)\eta^{\prime }\left(s\right)ds$) for every 0⩽s<t<∞ and every $0⩽\varphi \in C_{0}^{2}\left(R^{d}\right)$,

(48) $$\begin{array}{cc} & \int_{R^{d}}\varphi \left(x\right)\eta \left(\rho^{\varepsilon }\left(t,x\right)\right)dx-\int_{R^{d}}\varphi \left(x\right)\eta \left(\rho^{\varepsilon }\left(s,x\right)\right)dx\\ ⩽ & \int_{s}^{t}\int_{R^{d}}\mathrm{div}\left(b\left(x\right)\varphi \left(x\right)\right)q\left(\rho^{\varepsilon }\left(r,x\right)\right)dxdr+\frac{1}{2}\int_{s}^{t}\int_{R^{d}}\eta^{\prime \prime }\left(\rho^{\varepsilon }\left(r,x\right)\right)A^{2}\left(\rho^{\varepsilon }\right)\varphi \left(x\right)dxdr\\ & +\int_{s}^{t}dW_{r}\int_{R^{d}}\eta^{\prime }\left(\rho^{\varepsilon }\left(r,x\right)\right)A\left(\rho^{\varepsilon }\right)\varphi \left(x\right)dx+\varepsilon \int_{s}^{t}\int_{R^{d}}\eta \left(\rho^{\varepsilon }\left(r,x\right)\right)\Delta \varphi \left(x\right)dxdr,\;P-a.s. \end{array}$$

Furthermore, if one approaches ε↓0 in (48), then (5) holds for ρ(t,x). Thus, ρ is a stochastic entropy solution to (1).

**Step 3.** Existence of the stochastic strong entropy solution to the Cauchy problem (1).

For every ${\left\{F_{t}\right\}}_{t⩾0}$-adapted $L^{2}\left(R^{d}\right)$-valued stochastic process $\tilde{\rho }\left(t,x,\omega \right)$ (meeting (3) and (4)), every given $\psi \in C_{0}^{2}\left(R^{2d}\right)$ and every given smooth convex function η, we set $\tilde{\eta }$ by (6) and

$$S\left(\eta ,\psi \right)\left(s,t,v,y\right)=\int_{s}^{t}\int_{R^{d}}\eta \left(\tilde{\rho }\left(r,x\right)-v\right)A\left(\tilde{\rho }\left(r,x\right)\right)\psi \left(x,y\right)dxdW_{r},$$

then

$$\begin{array}{c} \int_{R^{d}}[\int_{s}^{t}\tilde{\eta }\left(r,v,y\right)dW_{r}{]}_{v=\rho^{\varepsilon }\left(t,y\right)}dy=\int_{R^{d}}S\left(\eta^{\prime },\psi \right)\left(s,t,\rho^{\varepsilon }\left(t,y\right),y\right)dy, \end{array}$$

where $\rho^{\varepsilon }$ is the unique solution of (22).

Let ϱ be given in (14), and set $\varrho_{\delta }\left(\cdot \right)=\varrho \left(\cdot /\delta \right)/\delta$, then for almost all ω∈Ω, we have

(49) $$\begin{array}{c} \int_{R^{d}}S\left(\eta^{\prime },\psi \right)\left(s,t,\rho^{\varepsilon }\left(t,y\right),y\right)dy=\lim_{\delta ↓0}\int_{R^{d}}\int_{R}S\left(\eta^{\prime },v,\psi \right)\left(s,t,v,y\right)\varrho_{\delta }\left(v-\rho^{\varepsilon }\left(t,y\right)\right)dvdy. \end{array}$$

In view of the Itô formula for semi-martingales (d(XY)=XdY+YdX+d[X,Y]), (49) and the integration by parts, one derives that

(50) $$\begin{array}{cc} & E\int_{R^{d}}S\left(\eta^{\prime },\psi \right)\left(s,t,\rho^{\varepsilon }\left(t,y\right),y\right)dy\\ = & E\int_{R^{d}}\int_{s}^{t}S\left(\eta^{\prime },\psi \right)\left(s,r,\rho^{\varepsilon }\left(r,y\right),y\right)drdy\\ & +E\int_{R^{d}}\int_{s}^{t}S\left(\eta^{\prime \prime },\psi \right)\left(s,r,\rho^{\varepsilon }\left(r,y\right),y\right)\left(-b\left(y\right)\cdot \nabla_{y}f\left(\rho^{\varepsilon }\left(r,y\right)\right)\right)drdy\\ & +E\int_{R^{d}}\int_{s}^{t}S\left(\eta^{\prime \prime },\psi \right)\left(s,r,\rho^{\varepsilon }\left(r,y\right),y\right)\varepsilon \Delta_{yy}\rho^{\varepsilon }\left(r,y\right)dr)dy\\ & +\frac{1}{2}E\int_{R^{d}}\int_{s}^{t}S\left(\eta^{\prime \prime \prime },\psi \right)\left(s,r,\rho^{\varepsilon }\left(r,y\right),y\right)A^{2}\left(\rho^{\varepsilon }\left(r,y\right)\right)drdy\\ & -E\int_{R^{d}}\int_{R^{d}}\int_{s}^{t}\eta^{\prime \prime }\left(\tilde{\rho }\left(r,x\right)-\rho^{\varepsilon }\left(r,y\right)\right)A\left(\tilde{\rho }\left(r,x\right)\right)A\left(\rho^{\varepsilon }\left(r,y\right)\right)\psi \left(x,y\right)drdxdy\\ =: & I_{\varepsilon }^{1}\left(s,t\right)+I_{\varepsilon }^{2}\left(s,t\right)+I_{\varepsilon }^{3}\left(s,t\right)+I_{\varepsilon }^{4}\left(s,t\right)+I_{\varepsilon }^{5}\left(s,t\right). \end{array}$$

The calculations for $I_{\varepsilon }^{i}\left(s,t\right)$ (i=1,2,3,4) are similar, and we take $I_{\varepsilon }^{2}\left(s,t\right)$ for a typical example. Firstly, through integration by parts, it follows that

(51) $$\begin{array}{cc} & |I_{\varepsilon }^{2}\left(s,t\right)|\\ = & \left|E\int_{R^{d}}\int_{s}^{t}{\left[\int_{s}^{r}\int_{R^{d}}\eta^{\prime \prime }\left(\tilde{\rho }\left(\tau ,x\right)-v\right)A\left(\tilde{\rho }\left(\tau ,x\right)\right){\mathrm{div}}_{y}\left(\psi \left(x,y\right)b\left(y\right)\right)dxdW_{\tau }\right]}_{v=\rho^{\varepsilon }\left(r,y\right)}f\left(\rho^{\varepsilon }\left(r,y\right)\right)drdy\right|\\ ⩽ & CE\int_{R^{d}}\int_{s}^{t}\underset{\left|v\right|⩽N_{1}}{\sup}|\int_{s}^{r}\int_{R^{d}}\eta^{\prime \prime }\left(\tilde{\rho }\left(\tau ,x\right)-v\right)A\left(\tilde{\rho }\left(\tau ,x\right)\right){\mathrm{div}}_{y}\left(\psi \left(x,y\right)b\left(y\right)\right)dxdW_{\tau }|drdy, \end{array}$$

where $N_{1}=N\vee {∥\rho_{0}∥}_{L^{\infty }}$.

For p>d∨2, using the Sobolev embedding theorem $W^{1,p}\left(-N_{1},N_{1}\right)\subset L^{\infty }\left(-N_{1},N_{1}\right)$ and Hölder inequality, from (51), we have

(52) $$\begin{array}{cc} & \underset{\varepsilon \to 0}{lim inf}I_{\varepsilon }^{2}\left(s,t\right)\\ ⩽ & C\int_{R^{d}}\int_{s}^{t}{\left(\int_{-N_{1}}^{N_{1}}E|\int_{s}^{r}\int_{R^{d}}\eta^{\prime \prime }\left(\tilde{\rho }\left(\tau ,x\right)-v\right)A\left(\tilde{\rho }\left(\tau ,x\right)\right){\mathrm{div}}_{y}\left(\psi \left(x,y\right)b\left(y\right)\right)dxdW_{\tau }{|}^{p}dv\right)}^{\frac{1}{p}}drdy\\ & +C\int_{R^{d}}\int_{s}^{t}{\left(\int_{-N_{1}}^{N_{1}}E|\int_{s}^{r}\int_{R^{d}}\eta^{\prime \prime \prime }\left(\tilde{\rho }\left(\tau ,x\right)-v\right)A\left(\tilde{\rho }\left(\tau ,x\right)\right){\mathrm{div}}_{y}\left(\psi \left(x,y\right)b\left(y\right)\right)dxdW_{\tau }{|}^{p}dv\right)}^{\frac{1}{p}}drdy\\ ⩽ & C\int_{R^{d}}\int_{s}^{t}{\left[\int_{-N_{1}}^{N_{1}}E{\left(\int_{s}^{r}|\int_{R^{d}}\eta^{\prime \prime }\left(\tilde{\rho }\left(\tau ,x\right)-v\right)A\left(\tilde{\rho }\left(\tau ,x\right)\right){\mathrm{div}}_{y}\left(\psi \left(x,y\right)b\left(y\right)\right)dx{|}^{2}d\tau \right)}^{\frac{p}{2}}dv\right]}^{\frac{1}{p}}drdy\\ & +C\int_{R^{d}}\int_{s}^{t}{\left[\int_{-N_{1}}^{N_{1}}E{\left(\int_{s}^{r}|\int_{R^{d}}\eta^{\prime \prime \prime }\left(\tilde{\rho }\left(\tau ,x\right)-v\right)A\left(\tilde{\rho }\left(\tau ,x\right)\right){\mathrm{div}}_{y}\left(\psi \left(x,y\right)b\left(y\right)\right)dx{|}^{2}d\tau \right)}^{\frac{p}{2}}dv\right]}^{\frac{1}{p}}drdy\\ ⩽ & C(N_{1}{,T,∥b∥}_{W^{1,\infty }},\eta ,\psi )\int_{s}^{t}{\left(r-s\right)}^{\frac{1}{2}}dr=C(N_{1}{,T,∥b∥}_{W^{1,\infty }}{,\eta ,\psi )|t-s|}^{\frac{3}{2}}=:D\left(s,t\right), \end{array}$$

where *D* is a deterministic function which meets the property (8).

By using dominated convergence theorem, we also have

(53) $$\begin{array}{c} \lim_{\varepsilon \to 0}I_{\varepsilon }^{5}\left(s,t\right)=-\int_{R^{d}}\int_{R^{d}}\int_{s}^{t}\eta^{\prime \prime }\left(\tilde{\rho }\left(r,x\right)-\rho \left(r,y\right)\right)A\left(\tilde{\rho }\left(r,x\right)\right)A\left(\rho \left(r,y\right)\right)\psi \left(x,y\right)drdxdy \end{array}$$

and

(54) $$\begin{array}{c} \lim_{\varepsilon \to 0}E\int_{R^{d}}S\left(\eta^{\prime },\psi \right)\left(s,t,\rho^{\varepsilon }\left(t,y\right),y\right)dy=E\int_{R^{d}}S\left(\eta^{\prime },\psi \right)\left(s,t,\rho \left(t,y\right),y\right)dy. \end{array}$$

Combining (50) and (52)–(54), we know that (7) is true for ρ.

(ii) In this case, we choose $\rho_{0}^{\varepsilon }\in BV\cap L^{\infty }\cap H^{1}\left(R^{d}\right)$ such that $\rho_{0}^{\varepsilon }\to \rho_{0}$ in $L^{2}\cap BV\left(R^{d}\right)$ as ε↓0. Let η:R→R be a $C^{\infty }$ even function satisfying

$$\begin{array}{c} \eta \left(0\right)=0,\;\;\eta^{\prime \prime }⩾0,\;\;\eta^{\prime }\left(r\right)=\left\{\begin{array}{cc} \;-1, & \mathrm{when}\;r<-1,\\ \in [-1,1], & \mathrm{when}\;|r|⩽1,\\ \;\;1, & \mathrm{when}\;r>1. \end{array}\right. \end{array}$$

For any δ>0, we define $\eta_{\delta }$ by $\eta_{\delta }\left(r\right)=\delta \eta \left(r/\delta \right)$. Then,

(55) $$\begin{array}{c} \eta_{\delta }\left(r\right)\to |r|\;\;\mathrm{as}\;\;\delta ↓0. \end{array}$$

Let $\varphi \left(x\right)=C_{1}e^{-|x|}$, with $C_{1}={\left[\int_{R^{d}}e^{-|x|}dx\right]}^{-1}$. Since $\rho^{\varepsilon }\in C\left(\left[0,T\right];L^{2}\left(\Omega ;H^{2}\left(R^{d}\right)\right)\right)$ for every T>0, we can take the derivative of (22) with respect to $x_{i}$ first, then by using the Itô formula to $\eta_{\delta }\left(\rho_{x_{i}}^{\varepsilon }\left(t,x\right)\right)$,

(56) $$\begin{array}{cc} & d\eta_{\delta }\left(\rho_{x_{i}}^{\varepsilon }\left(t,x\right)\right)+\eta_{\delta }^{\prime }\left(\rho_{x_{i}}^{\varepsilon }\left(t,x\right)\right)\partial_{x_{i}}\left(b\left(x\right)\cdot \nabla_{x}f\left(\rho^{\varepsilon }\left(t,x\right)\right)\right)dt\\ = & d\eta_{\delta }\left(\rho_{x_{i}}^{\varepsilon }\left(t,x\right)\right)+\eta_{\delta }^{\prime }\left(\rho_{x_{i}}^{\varepsilon }\left(t,x\right)\right)\partial_{x_{i}}b\left(x\right)\cdot \nabla_{x}f\left(\rho^{\varepsilon }\left(t,x\right)\right)dt\\ & +b\left(x\right)\cdot \nabla_{x}\left(\eta_{\delta }^{\prime }\left(\rho_{x_{i}}^{\varepsilon }\left(t,x\right)\right)f^{\prime }\left(\rho^{\varepsilon }\left(t,x\right)\right)\partial_{x_{i}}\rho^{\varepsilon }\left(t,x\right)\right)dt\\ & -\eta_{\delta }^{\prime \prime }\left(\rho_{x_{i}}^{\varepsilon }\left(t,x\right)\right)f^{\prime }\left(\rho^{\varepsilon }\left(t,x\right)\right)\partial_{x_{i}}\rho^{\varepsilon }\left(t,x\right)b\left(x\right)\cdot \nabla_{x}\rho_{x_{i}}^{\varepsilon }\left(t,x\right)dt\\ = & \varepsilon \eta_{\delta }^{\prime }\left(\rho_{x_{i}}^{\varepsilon }\left(t,x\right)\right)\Delta \rho_{x_{i}}^{\varepsilon }\left(t,x\right)dt+\eta_{\delta }^{\prime }\left(\rho_{x_{i}}^{\varepsilon }\left(t,x\right)\right)A^{\prime }\left(\rho^{\varepsilon }\left(t,x\right)\right)\rho_{x_{i}}^{\varepsilon }\left(t,x\right)dW_{t}\\ & +\frac{1}{2}\eta_{\delta }^{\prime \prime }\left(\rho_{x_{i}}^{\varepsilon }\left(t,x\right)\right){\left|A^{\prime }\left(\rho^{\varepsilon }\left(t,x\right)\right)\rho_{x_{i}}^{\varepsilon }\left(t,x\right)\right|}^{2}dt\\ = & \varepsilon \Delta \eta_{\delta }\left(\rho_{x_{i}}^{\varepsilon }\left(t,x\right)\right)dt+\eta_{\delta }^{\prime }\left(\rho_{x_{i}}^{\varepsilon }\left(t,x\right)\right)A^{\prime }\left(\rho^{\varepsilon }\left(t,x\right)\right)\rho_{x_{i}}^{\varepsilon }\left(t,x\right)dW_{t}\\ & +\frac{1}{2}\eta_{\delta }^{\prime \prime }\left(\rho_{x_{i}}^{\varepsilon }\left(t,x\right)\right){\left|A^{\prime }\left(\rho^{\varepsilon }\left(t,x\right)\right)\rho_{x_{i}}^{\varepsilon }\left(t,x\right)\right|}^{2}dt-\varepsilon \eta_{\delta }^{\prime \prime }\left(\rho_{x_{i}}^{\varepsilon }\left(t,x\right)\right){\left|\nabla_{x}\rho_{x_{i}}^{\varepsilon }\left(t,x\right)\right|}^{2}dt\\ ⩽ & \varepsilon \Delta \eta_{\delta }\left(\rho_{x_{i}}^{\varepsilon }\left(t,x\right)\right)dt+\eta_{\delta }^{\prime }\left(\rho_{x_{i}}^{\varepsilon }\left(t,x\right)\right)A^{\prime }\left(\rho^{\varepsilon }\left(t,x\right)\right)\rho_{x_{i}}^{\varepsilon }\left(t,x\right)dW_{t}\\ & +\frac{1}{2}\eta_{\delta }^{\prime \prime }\left(\rho_{x_{i}}^{\varepsilon }\left(t,x\right)\right){\left|A^{\prime }\left(\rho^{\varepsilon }\left(t,x\right)\right)\rho_{x_{i}}^{\varepsilon }\left(t,x\right)\right|}^{2}dt. \end{array}$$

Assume R>0, we set $\varphi_{R}\left(\cdot \right)=\varphi \left(\cdot /R\right)$, then

(57) $$\begin{array}{cc} & E\int_{R^{d}}\eta_{\delta }\left(\rho_{x_{i}}^{\varepsilon }\left(t,x\right)\right)\varphi_{R}\left(x\right)dx-\int_{R^{d}}\eta_{\delta }\left(\rho_{0,x_{i}}^{\varepsilon }\left(x\right)\right)\varphi_{R}\left(x\right)dx\\ ⩽ & \frac{1}{2}E\int_{0}^{t}\int_{R^{d}}\eta_{\delta }^{\prime \prime }\left(\rho_{x_{i}}^{\varepsilon }\left(r,x\right)\right){\left|A^{\prime }\left(\rho^{\varepsilon }\left(r,x\right)\right)\rho_{x_{i}}^{\varepsilon }\left(r,x\right)\right|}^{2}\varphi_{R}\left(x\right)dxdr\\ & +\frac{\varepsilon C}{R^{2}}E\int_{0}^{t}\int_{R^{d}}\eta_{\delta }\left(\rho_{x_{i}}^{\varepsilon }\left(r,x\right)\right)\varphi_{R}\left(x\right)dxdr\\ & +{C(∥b∥}_{W^{1,\infty }\left(R^{d}\right)},∥f^{\prime }{∥}_{L^{\infty }\left(R\right)})E\int_{0}^{t}\int_{R^{d}}\left|\eta_{\delta }^{\prime \prime }\left(\rho_{x_{i}}^{\varepsilon }\left(r,x\right)\right)\right|\left|\rho_{x_{i}}^{\varepsilon }\left(r,x\right)\right|\left|\nabla_{x}\rho_{x_{i}}^{\varepsilon }\left(r,x\right)\right|\varphi_{R}\left(x\right)dxdr\\ & +{C(∥b∥}_{W^{1,\infty }\left(R^{d}\right)},∥f^{\prime }{∥}_{L^{\infty }\left(R\right)})E\int_{0}^{t}\int_{R^{d}}\left|\nabla_{x}\rho^{\varepsilon }\left(r,x\right)\right|\varphi_{R}\left(x\right)dxdr\\ ⩽ & CE\int_{0}^{t}\int_{R^{d}}\left|\rho_{x_{i}}^{\varepsilon }\left(r,x\right)\right|1_{|\rho_{x_{i}}^{\varepsilon }\left(r,x\right)|⩽\delta }\varphi_{R}\left(x\right)dxdr+\frac{\varepsilon C}{R^{2}}E\int_{0}^{t}\int_{R^{d}}\eta_{\delta }\left(\rho_{x_{i}}^{\varepsilon }\left(r,x\right)\right)\varphi_{R}\left(x\right)dxdr\\ & +C\left[E\int_{0}^{t}\int_{R^{d}}\frac{1}{\delta }\left|\rho_{x_{i}}^{\varepsilon }\left(r,x\right)\right|1_{|\rho_{x_{i}}^{\varepsilon }\left(r,x\right)|⩽\delta }\left|\nabla_{x}\rho_{x_{i}}^{\varepsilon }\left(r,x\right)\right|\varphi_{R}\left(x\right)dxdr\right]\\ & +CE\int_{0}^{t}\int_{R^{d}}\left|\nabla_{x}\rho^{\varepsilon }\left(r,x\right)\right|\varphi_{R}\left(x\right)dxdr, \end{array}$$

where, in the last inequality, we apply the fact $\eta_{\delta }^{\prime \prime }\left(\rho_{x_{i}}^{\varepsilon }\left(r,x\right)\right)⩽C1_{|\rho_{x_{i}}^{\varepsilon }\left(r,x\right)|⩽\delta }/\delta$.

Observing that, for almost everywhere, $\left(t,x\right)\in \left[0,T\right]\times R^{d}$, $|\rho_{x_{i}}^{\varepsilon }|1_{|\rho_{x_{i}}^{\varepsilon }|⩽\delta }/\delta \to 0$ almost surely, as δ↓0, from (57) by using dominated convergence theorem, if one lets δ↓0 first and sums over *i* from 1 to *d* next,

$$\begin{array}{cc} & E\int_{R^{d}}|\nabla \rho^{\varepsilon }\left(t,x\right)|\varphi_{R}\left(x\right)dx-\int_{R^{d}}\left|\nabla \rho_{0}^{\varepsilon }\left(x\right)\right|\varphi_{R}\left(x\right)dx\\ ⩽ & \frac{\varepsilon C}{R^{2}}E\int_{0}^{t}\int_{R^{d}}|\nabla \rho^{\varepsilon }\left(r,x\right)|\varphi_{R}\left(x\right)dxdr+CE\int_{0}^{t}\int_{R^{d}}\left|\nabla_{x}\rho^{\varepsilon }\left(r,x\right)\right|\varphi_{R}\left(x\right)dxdr. \end{array}$$

Therefore,

(58) $$\begin{array}{c} \underset{0⩽t⩽T}{\sup}E\int_{R^{d}}|\nabla \rho^{\varepsilon }{\left(t,x\right)|dx⩽C(∥b∥}_{W^{1,\infty }\left(R^{d}\right)},∥f^{\prime }{∥}_{L^{\infty }\left(R\right)},\varepsilon )\int_{R^{d}}\left|\nabla \rho_{0}^{\varepsilon }\left(x\right)\right|dx. \end{array}$$

Let $\eta_{\delta }$ be defined as before (meeting property (55)), and φ(x)=1 when |x|⩽1. We multiply $\varphi_{R}$ on both sides of (56), in view of integration by parts, we derive that

(59) $$\begin{array}{cc} & E\int_{R^{d}}\eta_{\delta }\left(\rho_{x_{i}}^{\varepsilon }\left(t,x\right)\right)dx-\int_{R^{d}}\eta_{\delta }\left(\rho_{0,x_{i}}^{\varepsilon }\left(x\right)\right)dx\\ ⩽ & \frac{1}{2}E\int_{0}^{t}\int_{R^{d}}\eta_{\delta }^{\prime \prime }\left(\rho_{x_{i}}^{\varepsilon }\left(r,x\right)\right){\left|A^{\prime }\left(\rho^{\varepsilon }\left(r,x\right)\right)\rho_{x_{i}}^{\varepsilon }\left(r,x\right)\right|}^{2}\varphi_{R}\left(x\right)dxdr\\ & +\frac{\varepsilon }{R^{2}}E\int_{0}^{t}\int_{R^{d}}\Delta \varphi \left(\frac{x}{R}\right)\eta_{\delta }\left(\rho_{x_{i}}^{\varepsilon }\left(r,x\right)\right)dxdr\\ & -E\int_{0}^{t}\int_{R^{d}}\eta_{\delta }^{\prime }\left(\rho_{x_{i}}^{\varepsilon }\left(r,x\right)\right)\partial_{x_{i}}b\left(x\right)\cdot \nabla_{x}f\left(\rho^{\varepsilon }\left(r,x\right)\right)\varphi \left(\frac{x}{R}\right)dxdr\\ & +E\int_{0}^{t}\int_{R^{d}}\eta_{\delta }^{\prime }\left(\rho_{x_{i}}^{\varepsilon }\left(r,x\right)\right)f^{\prime }\left(\rho^{\varepsilon }\left(r,x\right)\right)\partial_{x_{i}}\rho^{\varepsilon }\left(r,x\right){\mathrm{div}}_{x}\left(b\left(x\right)\varphi \left(\frac{x}{R}\right)\right)dxdr\\ & -E\int_{0}^{t}\int_{R^{d}}\eta_{\delta }^{\prime \prime }\left(\rho_{x_{i}}^{\varepsilon }\left(r,x\right)\right)f^{\prime }\left(\rho^{\varepsilon }\left(r,x\right)\right)\partial_{x_{i}}\rho^{\varepsilon }\left(r,x\right)b\left(x\right)\cdot \nabla_{x}\rho_{x_{i}}^{\varepsilon }\left(r,x\right)\varphi \left(\frac{x}{R}\right)dxdr\\ ⩽ & CE\int_{0}^{t}\int_{R^{d}}\left|\rho_{x_{i}}^{\varepsilon }\left(r,x\right)\right|1_{|\rho_{x_{i}}^{\varepsilon }\left(r,x\right)|⩽\delta }\varphi_{R}\left(x\right)dxdr+\frac{\varepsilon }{R^{2}}E\int_{0}^{t}\int_{R^{d}}\Delta \varphi \left(\frac{x}{R}\right)\eta_{\delta }\left(\rho_{x_{i}}^{\varepsilon }\left(r,x\right)\right)dxdr\\ & +{C(∥b∥}_{W^{1,\infty }\left(R^{d}\right)},∥f^{\prime }{∥}_{L^{\infty }\left(R\right)})E\int_{0}^{t}\int_{R^{d}}\left|\nabla_{x}\rho^{\varepsilon }\left(r,x\right)\right|\left[\varphi \left(\frac{x}{R}\right)+\frac{1}{R}\left|\nabla \varphi \left(\frac{x}{R}\right)\right|\right]dxdr\\ & +{C(∥b∥}_{L^{\infty }\left(R^{d}\right)},∥f^{\prime }{∥}_{L^{\infty }(R})E\int_{0}^{t}\int_{R^{d}}\frac{1}{\delta }\left|\rho_{x_{i}}^{\varepsilon }\left(r,x\right)\right|1_{|\rho_{x_{i}}^{\varepsilon }\left(r,x\right)|⩽\delta }\left|\nabla_{x}\rho_{x_{i}}^{\varepsilon }\left(r,x\right)\right|\varphi \left(\frac{x}{R}\right)dxdr. \end{array}$$

With the help of (25) and (58), from (59), by taking δ↓0 first, R↑∞ next, then

(60) $$\begin{array}{c} \underset{0⩽t⩽T}{\sup}E\int_{R^{d}}|\nabla \rho^{\varepsilon }{\left(t,x\right)|dx⩽C(∥b∥}_{W^{1,\infty }\left(R^{d}\right)},∥f^{\prime }{∥}_{L^{\infty }\left(R\right)})\int_{R^{d}}\left|\nabla \rho_{0}^{\varepsilon }\left(x\right)\right|dx. \end{array}$$

From (24) and (60), and noting that $\rho_{0}^{\varepsilon }\to \rho_{0}$ in $L^{2}\cap BV\left(R^{d}\right)$, by letting ε↓0, (12) is true and we finish the proof. □

## 4. Proof of Theorem 3

For ε>0, we denote $\rho^{\varepsilon }$ the unique solution of (22) with $\rho_{0}^{\varepsilon }\in L^{\infty }\cap BV\cap H^{1}\left(R^{d}\right)$ and $\rho_{0}^{\varepsilon }\to \rho_{0}\in L^{2}\cap BV\left(R^{d}\right)$, as ε↓0. Let ${\tilde{\rho }}_{0}^{\varepsilon }\in L^{1}\cap L^{\infty }\cap H^{1}\left(R^{d}\right)$ and ${\tilde{\rho }}_{0}^{\varepsilon }\to {\tilde{\rho }}_{0}$ in $L^{1}\cap L^{2}\left(R^{d}\right)$, as ε↓0. We assume ${\tilde{\rho }}^{\varepsilon }$ is the unique stochastic strong entropy solution of the following Cauchy problem:

(61) $$\begin{array}{c} \left\{\begin{array}{c} d{\tilde{\rho }}^{\varepsilon }\left(t,x\right)+\tilde{b}\left(x\right)\cdot \nabla_{x}\tilde{f}\left({\tilde{\rho }}^{\varepsilon }\left(t,x\right)\right)dt=\varepsilon \Delta {\tilde{\rho }}^{\varepsilon }\left(t,x\right)dt+A\left({\tilde{\rho }}^{\varepsilon }\left(t,x\right)\right)dW_{t},\;\;t>0,\;x\in R^{d},\\ {\tilde{\rho }}^{\varepsilon }{\left(t,x\right)|}_{t=0}={\tilde{\rho }}_{0}^{\varepsilon }\left(x\right),\;x\in R^{d}. \end{array}\right. \end{array}$$

Let $\eta_{\delta }$ be given by (56). We set the difference $\rho^{\varepsilon }\left(t,x\right)-{\tilde{\rho }}^{\varepsilon }\left(t,x\right)$ by $\xi^{\varepsilon }\left(t,x\right)$. Since $\rho_{0}^{\varepsilon },{\tilde{\rho }}_{0}^{\varepsilon }\in H^{1}\left(R^{d}\right)$, $\xi^{\varepsilon }\in L^{2}\left(\left[0,T\right]\times \Omega ;H^{2}\left(R^{d}\right)\right)$. From (22) and (61) and by applying Itô’s formula, then

(62) $$\begin{array}{ccc} d\eta_{\delta }\left(\xi^{\varepsilon }\left(t,x\right)\right) & = & -\eta_{\delta }^{\prime }\left(\xi^{\varepsilon }\left(t,x\right)\right)[b\left(x\right)\cdot \nabla_{x}f\left(\rho^{\varepsilon }\left(t,x\right)\right)-\tilde{b}\left(x\right)\cdot \nabla \tilde{f}\left({\tilde{\rho }}^{\varepsilon }\left(t,x\right)\right)dt\\ & & +\varepsilon \eta_{\delta }^{\prime }\left(\xi^{\varepsilon }\left(t,x\right)\right)\Delta \xi^{\varepsilon }\left(t,x\right)dt+\frac{1}{2}\eta_{\delta }^{\prime \prime }\left(\xi^{\varepsilon }\left(t,x\right)\right){\left|A\left(\rho^{\varepsilon }\left(t,x\right)\right)-A\left({\tilde{\rho }}^{\varepsilon }\left(t,x\right)\right)\right|}^{2}dt\\ & & +\eta_{\delta }^{\prime }\left(\xi^{\varepsilon }\left(t,x\right)\right)\left[A\left(\rho^{\varepsilon }\left(t,x\right)\right)-A\left({\tilde{\rho }}^{\varepsilon }\left(t,x\right)\right)\right]dW_{t}\\ & ⩽ & -\eta_{\delta }^{\prime }\left(\xi^{\varepsilon }\left(t,x\right)\right)\left[b\left(x\right)-\tilde{b}\left(x\right)\right]\cdot \nabla_{x}f\left(\rho^{\varepsilon }\left(t,x\right)\right)dt\\ & & -\eta_{\delta }^{\prime }\left(\xi^{\varepsilon }\left(t,x\right)\right)\tilde{b}\left(x\right)\cdot \nabla \left[f\left(\rho^{\varepsilon }\left(t,x\right)\right)-\tilde{f}\left(\rho^{\varepsilon }\left(t,x\right)\right)\right]dt\\ & & -\eta_{\delta }^{\prime }\left(\xi^{\varepsilon }\left(t,x\right)\right)\tilde{b}\left(x\right)\cdot \nabla \left[\tilde{f}\left(\rho^{\varepsilon }\left(t,x\right)\right)-\tilde{f}\left({\tilde{\rho }}^{\varepsilon }\left(t,x\right)\right)\right]dt\\ & & +\varepsilon \Delta \eta_{\delta }\left(\xi^{\varepsilon }\left(t,x\right)\right)dt+\eta_{\delta }^{\prime }\left(\xi^{\varepsilon }\left(t,x\right)\right)\left[A\left(\rho^{\varepsilon }\left(t,x\right)\right)-A\left({\tilde{\rho }}^{\varepsilon }\left(t,x\right)\right)\right]dW_{t}\\ & & +\frac{1}{2}\eta_{\delta }^{\prime \prime }\left(\xi^{\varepsilon }\left(t,x\right)\right){\left|A\left(\rho^{\varepsilon }\left(t,x\right)\right)-A\left({\tilde{\rho }}^{\varepsilon }\left(t,x\right)\right)\right|}^{2}dt. \end{array}$$

Let $\varphi_{R}$ be given in (57) and we integrate (62) against $\varphi_{R}$. By analogue calculations from (56) to (59), and then letting δ↓0 first, R↑∞ next, it yields that

$$\begin{array}{c} E\int_{R^{d}}\left|\xi^{\varepsilon }\left(t,x\right)\right|dx⩽\int_{R^{d}}\left|\xi^{\varepsilon }\left(0,x\right)\right|dx+C∥\mathrm{div}\tilde{b}{∥}_{L^{\infty }\left(R^{d}\right)}∥{\tilde{f}}^{\prime }{∥}_{L^{\infty }\left(R\right)}E\int_{0}^{t}\int_{R^{d}}\left|\xi^{\varepsilon }\left(r,x\right)\right|dxdr\\ +C\left[∥b-\tilde{b}{∥}_{L^{\infty }\left(R^{d}\right)}∥f^{\prime }{∥}_{L^{\infty }\left(R\right)}+∥\tilde{b}{∥}_{L^{\infty }\left(R^{d}\right)}{∥f^{\prime }-{\tilde{f}}^{\prime }∥}_{L^{\infty }\left(R\right)}\right]E\int_{0}^{t}\int_{R^{d}}\left|\nabla \rho^{\varepsilon }\left(r,x\right)\right|dxdr. \end{array}$$

With the help of (60), then

(63) $$\begin{array}{c} E\int_{R^{d}}\left|\xi^{\varepsilon }\left(t,x\right)\right|dx⩽\int_{R^{d}}\left|\xi^{\varepsilon }\left(0,x\right)\right|dx+C∥\mathrm{div}\tilde{b}{∥}_{L^{\infty }\left(R^{d}\right)}∥{\tilde{f}}^{\prime }{∥}_{L^{\infty }\left(R\right)}E\int_{0}^{t}\int_{R^{d}}\left|\xi^{\varepsilon }\left(r,x\right)\right|dxdr\\ +C\left({∥b∥}_{W^{1,\infty }\left(R^{d}\right)},∥f^{\prime }{∥}_{L^{\infty }\left(R\right)},{∥\tilde{b}∥}_{L^{\infty }\left(R^{d}\right)}\right)\left[∥b-\tilde{b}{∥}_{L^{\infty }\left(R^{d}\right)}+{∥f^{\prime }-{\tilde{f}}^{\prime }∥}_{L^{\infty }\left(R\right)}\right]\int_{R^{d}}\left|\nabla \rho_{0}^{\varepsilon }\right|dx. \end{array}$$

From (63), there is a constant C>0, which is dependent on ${∥b∥}_{W^{1,\infty }\left(R^{d}\right)}$, ${∥f^{\prime }∥}_{L^{\infty }\left(R\right)}$, $∥{\tilde{f}}^{\prime }{∥}_{L^{\infty }\left(R\right)}$, ${∥\mathrm{div}\tilde{b}∥}_{L^{\infty }\left(R^{d}\right)}$, ${∥\tilde{b}∥}_{L^{\infty }\left(R^{d}\right)}$ and *T*, such that

(64) $$\begin{array}{c} E\int_{R^{d}}\left|\xi^{\varepsilon }\left(t,x\right)\right|dx⩽\int_{R^{d}}\left|\xi^{\varepsilon }\left(0,x\right)\right|dx+C\left[∥b-\tilde{b}{∥}_{L^{\infty }\left(R^{d}\right)}+{∥f^{\prime }-{\tilde{f}}^{\prime }∥}_{L^{\infty }\left(R\right)}\right]\int_{R^{d}}\left|\nabla \rho_{0}^{\varepsilon }\left(x\right)\right|dx. \end{array}$$

From (64), by taking ε↓0, one ends up with the inequality (13). □

## 5. Conclusions

In this paper, we have established three results on the existence and uniqueness of stochastic entropy solutions for a nonlinear transport equation by a stochastic perturbation, and the continuous dependence of stochastic strong entropy solutions on the coefficient *b* and the nonlinear function *f*. Compared with the results on uniqueness given in [11,17], Theorem 1 is new since the 1/2-Hölder continuity of *A* is enough to ensure the uniqueness, and compared with the results on uniqueness for stochastic differential equations in [32], the hypotheses of 1/2-Hölder continuity on *A* is optimal. Moreover, we develop a new method of parabolic approximation to obtain the existence of solutions, which sheds some new light on the method of vanishing viscosity put forth by Feng and Nualart [11]. 
